# Evaluation of potentially inappropriate medications use and medication complexity in elderly patients applying to community pharmacy in Turkey

**DOI:** 10.1186/s12877-023-04381-4

**Published:** 2023-10-13

**Authors:** Aslınur Albayrak, Halil Demirbaş

**Affiliations:** 1 Department of Clinical Pharmacy, Faculty of Pharmacy, Suleyman Demirel University, Isparta, Turkey; 2 Faculty of Pharmacy, Suleyman Demirel University, Isparta, Turkey

**Keywords:** Beers criteria, Elderly, Medication regimen complexity, Potentially inappropriate medications

## Abstract

**Background:**

Older adults often use multiple medicines to manage comorbidities well or to prevent associated complications. This study aims to determine polypharmacy, the use of potentially inappropriate medications (PIMs) using the 2019 Beers Criteria and to determine the Medication Regimen Complexity Index (MRCI) score. It also aims to identify factors associated with the presence of PIMs and the MRCI score.

**Methods:**

This cross-sectional study was carried out between 6 and 2023 and 5 May 2023 in a community pharmacy in Turkey. Elderly patients over 65 years of age, who used at least one drug, and who came to the pharmacy for any reason were included in the study. PIMs were determined according to the 2019 Beers Criteria. The Turkish validated version of the MRCI was used to determine the medication complexity score.

**Results:**

200 patients were included in this study. 59.5% of the patients were female and the median age was 70 (IQR, 66-74.75). Polypharmacy was detected in 33% of patients. The use of PIMs was determined in 63.5% of the patients. The median of the MRCI score was 11 (IQR, 7–15). The number of chronic diseases and drugs, presence of polypharmacy, MRCI score and mental disorders were found to be significantly higher in those with PIMs than in those without (p < 0.05). Having less than eight years of education, presence of polypharmacy, the presence of comorbidity (diabetes mellitus, cardiovascular disease, thyroid, chronic obstructive pulmonary disease (COPD), asthma and mental disorders) were associated with significantly higher MRCI scores (p < 0.05).

**Conclusions:**

According to the results of our study, it was found that the elderly patients who came to the pharmacy had low MRCI scores, but had high PIMs use. Community pharmacists have an important role in identifying inappropriate drug use, so they should be trained to develop skills in identifying and reducing PIMs in older patients.

## Background

Older adults often have multimorbidities, so they use multiple medicines to manage these comorbidities or to prevent associated complications [1]. Multiple medicine use, commonly referred to as polypharmacy, is associated with adverse outcomes such as adverse drug reactions, falls, prolonged hospital stay, death, and hospital readmission soon after discharge [2].

Polypharmacy is strongly associated with potentially inappropriate medications (PIMs) [3]. There are two types of criteria, explicit and implicit, for the detection of PIMs. Implicit PIM criteria are judgmental quality indicators that focus on the patient rather than drugs or diseases. Although patient-centred, it is time consuming and largely dependent on the knowledge and experience of the prescriber. Explicit criteria are established based on literature review, expert opinions, and consensus opinion [4]. The Beers Criteria is one of the oldest and most widely used explicit criteria [4, 5]. The Beers Criteria was first developed in 1991 by Dr. Mark Beers and updated by the American Geriatrics Society every three years since 2012. Beers criteria have been updated as of 2015, 2019 and last May 2023 [6, 7]. The Beers Criteria include recognizing PIMs and providing safer alternatives where applicable PIM use can result from overprescribing, underprescribing and wrong prescribing [4].The PIM use also increases the incidence of drug-related problems, and adverse drug reactions. [8, 9]. It also negatively affects the patient’s quality of life [10]. This tool is for clinicians to manage and improve prescribing practice in older adults. Clinicians can use this list to review their patients’ medications, when prescribing a new medication, or for their hospitalized or outpatient patients [11]. Thus, they can prevent the use of PIM and its negative consequences. In a systematic review, the mean prevalence of PIM use according to Beers Criteria was 65% in a total of 221,879 older adults. For the gastrointestinal tract, the mean prevalence of PIMs was 15.3% and the mean prevalence of proton pump inhibitors was 27.7% [9]. Factors related to PIM use vary. In a systematic review examining factors associated with PIM use in primary care, more medication use and a higher number of comorbidities were found to be risk factors associated with PIM [12]. In other studies, certain chronic diseases, such as diabetes, hypertension, depression, osteoporosis, have been associated with higher PIM use compared to older adults without these chronic conditions [13, 14].

Polypharmacy in older adults brings with it complex drug regimens [15, 16]. Older adults have decreased sensory and cognitive functions. This can lead to medication errors and medication-related problems [17, 18]. To reduce these problems, the medication regimen should be simplified [19]. There are many tools available for medication regimen complexity. The first of these was the ‘‘Medication Complexity Index’’ (MCI) by Kelley et al. in 1988 [20]. Subsequently, ‘‘The Epilepsy Drug and Treatment Complexity Index’’ (EMTCI) was developed by Dilorio et al. in 2003 [21]. In addition, “Antiretroviral Medication Complexity Index” (AMCI) was developed by Dilorio et al. [22]. In 2004, the “Medication Regimen Complexity Index” (MRCI) scale was developed by George et al. through reviews of the literature and expert panel [23]. This tool is the most common, reliable and verified [19]. The MRCI is a 65-item scale consisting of three parts that assess dosage forms, dosage frequency, and additional directions for use. It was validated in Turkish by Okuyan et al. [24]. Both the Beers Criteria and the MRCI tool aim to simplify drug regimens [19]. Studies were showing that medication regimen complexity was associated with parameters such as hospital readmission and drug adherence [25–28]. While studies showed MRCI scores were positively correlated with the number of medications, no correlation was found with age and gender [23, 24]. Number of comorbidities, comorbidities such as chronic pulmonary disease, diabetes, and congestive heart failure, prevalence of self-reported pain were also associated with higher MRCI scores [29, 30].

It is important to measure the polypharmacy, PIM and MRCI scores of patients applying to the pharmacy and to raise the awareness of community pharmacists on this issue. Because community pharmacists have an important role to play in determining PIM use and medication complexity [19]. By educating patients, they can reduce inappropriate drug use and polypharmacy. They can also contribute to simplifying drug regimens [19, 31].

The elderly population is increasing worldwide and Turkey is one of these countries [32]. According to the 2022 report of the Turkish Statistical Institute, while the ratio of the elderly population to the total population in Turkey was 8.5% in 2017, it increased to 9.9% in 2022. In Isparta province, the ratio of the elderly population to the total population in 2022 is 13.9%, which is higher than the average of Turkey [33]. Therefore, this study aims to determine polypharmacy, PIM use and MRCI scores of elderly patients applying to the pharmacy in Isparta. It also aims to determine factors associated with the presence of PIMs and the MRCI score.

## Method

### Study design and setting

This cross-sectional study was carried out between 6 and 2023 and 5 May 2023 in a community pharmacy in Isparta, Turkey. Elderly patients over 65 years of age, who used at least one drug, and who came to the pharmacy for any cause were incorporated in the study. Each patient was evaluated only once. The research was conducted in a randomly selected pharmacy in Isparta, close to a primary health care center.

### Ethics approval and consent to participate

#### Ethical approval

of the study was obtained from the Suleyman Demirel Clinical Research Ethics Committee (Approval No: 196 Date: 07.07.2022). An informed consent form was obtained from all participants. The pharmacist was also informed about the study and permission was obtained.

### Sample Size

With the Raosoft sample size calculator, when the population size was unknown, the sample size was calculated as 377 with a 5% margin of error, 95% confidence interval and 50% distribution rate [34]. Convenience sampling method was used.

### Data collection

Data were collected by a senior pharmacy student (5th grade) from only one pharmacy, four days a week, through face-to-face interviews with patients.

A clinical pharmacist faculty member supervised the study. Data collection training was given to the senior pharmacy student, and how to communicate with the patients and how to calculate the scores was explained. PIMs and MRCI scores were also rechecked by the clinical pharmacist.

The characteristics of the participants, such as age, gender, allergy history, educational status, comorbidities, and hospitalization history in the last six months, were collected in a special form. In addition, the form included the indication, dosage and special instructions for use, if any, whether there was PIM or not, from which drug and the reason, and MRCI scores.

### Measures

#### Polypharmacy

Although there is no specific definition of polypharmacy, it is generally accepted as the use of five or more drugs [35]. In this study, polypharmacy was defined as the use of five or more drugs.

#### Potentially inappropriate medications (PIMs)

PIMs were determined according to the 2019 Beers Criteria of the American Geriatrics Society (AGS), as the latest Beers Criteria were the Beers 2019 criteria during our study [5]. The Beers Criteria are a list of drugs that should be avoided by older adults in most cases or for a particular disease or condition. The Beers Criteria fall into five categories: (1) PIMs in older adults; (2) PIMs to avoid in older adults with certain conditions; (3) medications that should be used with caution in older adults; (4) clinically important drug-drug interactions to avoid and (5) medications that should be avoided or given in different doses for those with impaired renal function. Patients using any of the potentially inappropriate drugs listed in the Beers 2019 Criteria were assigned to the PIM use group, and otherwise to the no PIM use group.

#### Medication regimen complexity index (MRCI)

The MRCI scale was developed by George et al. [23] and validated in Turkish by Okuyan et al. [24]. The Turkish version of MRCI was used in our study and necessary permissions were obtained from the authors. The medication regimen complexity scale consists of 65 items. Among 65 items, there is information about dosage forms, dose frequencies, and drug administration. The scale consists of three sections. Section A covers dosage forms, section B relates to dose frequency, and section C contains additional instructions for drug administration. Section A was divided into sections according to the routes of administration as oral, topical, inhaler, ear, nose, eye. Each dosage form was scored differently, with higher scores being given as the dosage form became more complex. For example, in the oral section, the dosage form was 1 point for the capsule and tablet, while the sublingual tablet scored 2 points. In the inhaler section, for example the aeroliser scored 3 points, while the nebulizers scored 5 points. In section B, it is scored once a day with 1 point, every 12 h with 2.5 points, and every 8 h with 3.5 points. In section C, for example, if it needs to be taken at the specified time in the treatment (at 8 o’clock in the morning), it is scored with 1 point, if it is related to food (before, after, or with a meal) it is scored with 1 point, and if it needs to be taken with a specific liquid, it is scored with 1 point. MRCI A, B and C scores were calculated for all medicines used by each patient. A, B, and C scores were then summed to calculate the total MRCI score. MRCI scores start at 0 for a patient not taking medication and there is no upper limit. A high MRCI score indicates complex treatments.

### Statistical analysis

SPSS software (version 20) was used for data analysis. Variables were expressed as mean ± standard deviation, median (interquartile range), and percentage. The Chi-square test was used to compare categorical variables. Student’s T-test and Mann-Whitney U tests were used to analyze continuous independent variables with two groups. The Kruskal-Wallis test was used to analyze the significant difference between continuous variables of three or more independent groups. P values less than 0.05 were considered statistically significant.

## Results

200 patients were included in this study. 59.5% of the patients were female and the median age was 70 (IQR, 66-74.75). The median of the total number of drugs used was 4 (IQR, 3–5). Polypharmacy was detected in 33% of patients. 24% of the patients had a history of hospitalization within the last six months. The most common comorbidities are cardiovascular diseases (88%) and diabetes (33.5%). Table 1 shows the socio-demographic and clinical characteristics of the patients.

**Table 1:** Socio-demographic and clinical characteristics of the patients

Characteristics	
**Gender (%)**	
Female	119 (59.5)
Male	81 (40.5)
**Age years median (IQR)**	70 (66-74.75)
Education level*	
< 8 years	113 (56.5)
≥ 8 years	87 (43.5)
**Marital status (%)**	
Single	8 (4)
Married	144 (72)
Widow	48 (24)
**Drug allergy (%)**	
Yes	13 (6.5)
No	187 (93.5)
**Number of drugs (median, IQR)**	4 (3–5)
**Polypharmacy (Concurrent use of ≥ 5 drugs)**	67 (33.5)
**History of hospitalization in the last 6 months (%)**	
Yes	48 (24)
No	152 (76)
**Number of comorbidities (median, IQR)**	2 (1–3)
**Comorbidities- ICD 10 (%)**	
**E00-E89 Endocrine, nutritional and metabolic diseases**	76 (38)
E00-E07 Disorders of thyroid gland	18 (9)
E08-E13 Diabetes mellitus	67 (33.5)
**F01-F99 Mental, Behavioral and Neurodevelopmental disorders**	47 (23.5)
**I00–I99 Diseases of the circulatory system**	176 (88)
**J00–J99 Diseases of the respiratory system**	44 (22)
J 44 COPD	15 (75)
J 45 Asthma	30 (15)
**N00–N99 Diseases of the genitourinary system**	27 (13.5)
N18 Chronic Kidney Disease	27 (13.5)
**MRCI score** **(mean ± SD)** **(median, IQR)** **(min-max)**	11.54 ± 6.027 11 (7–15) (2–33)
MRCI Part A (mean ± SD)	4.63 ± 2.64
MRCI Part B (mean ± SD)	4.77 ± 2.48
MRCI Part C (mean ± SD)	2.13 ± 1.28

* The minimum period of compulsory education in Turkey is 8 years. ICD10 International Statistical Classification of Diseases and Related Health Problems 10th Revision, IQR: Interquartile range, SD: Standard deviation, COPD: Chronic obstructive pulmonary disease, MRCI: Medication Regimen Complexity Index

### Potentially inappropriate medications (PIMs)

In the study, the use of PIMs was determined in 63.5% of the patients. The use of PIMs, which should be avoided in 29% of the patients and should be used with caution in 47%, was detected. No PIMs related to avoidance or dose adjustment in renal dysfunction and PIMs related to clinically significant drug-drug interactions were found. Table 2 shows the frequency of use of PIMs by patients.

**Table 2:** Frequency of PIMs use in the study population

Presence of PIMs	Frequency (%)
Yes	127 (63.5)
No	73 (36.5)
**Numbers of PIMs**	
One PIM	76 (38)
Two PIMs	49 (24.5)
Three or more PIMs	2 (1)
**PIMs “That Should Be Avoided”**	
Yes	58 (29)
No	142 (71)
**Numbers of PIMs Use ‘‘That Should Be Avoided’’**	
One PIM	55 (27.5)
Two PIMs	3 (1.5)
**PIMs “That Should Be Used With Caution”**	
Yes	94 (47)
No	106 (53)
**Numbers of PIMs Use With Caution**	
One PIM	71 (35.5)
Two PIM	22 (11)
Three or more PIMs	1 (0.5)

PIMs: Potantially inappropriate medications

**Fig. 1:**
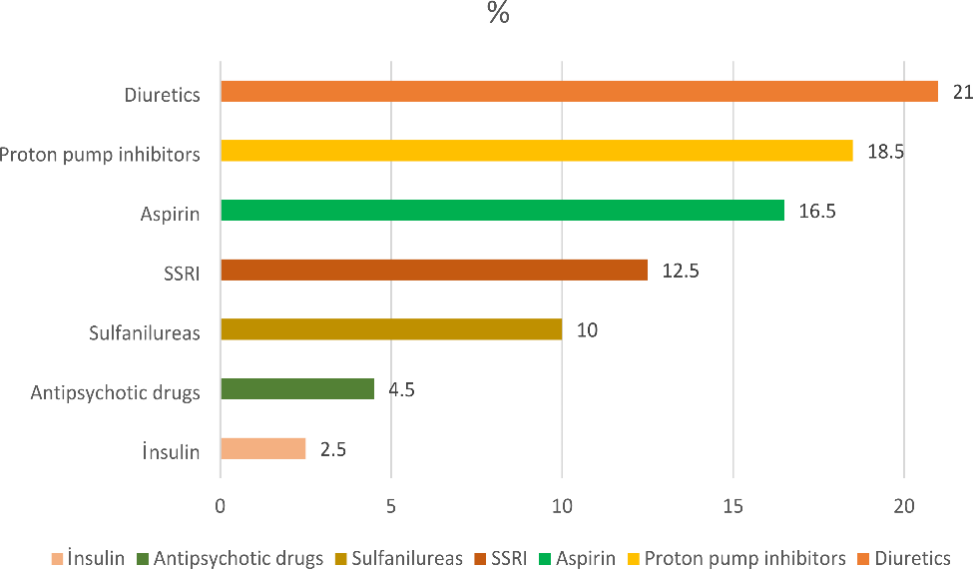
Most common PIMs examples

The most common examples of PIMs from patients were diuretics (21%), proton pump inhibitors (18.5%), and aspirin (16.5%) (Fig. 1).

Table 3 shows the presence of PIMs compared with demographic and clinical characteristics. The number of chronic diseases and drugs, presence of polypharmacy, MRCI score, mental disorders were found to be significantly higher in those with PIMs than in those without (p < 0.05). Gender, age, education level, marital status, drug allergy, and hospitalization history in the last six months did not differ significantly in terms of the presence of PIM (p > 0.05).

**Table 3:** Comparison of PIMs presence with demographic and clinical characteristics

Characteristics	PIMs Use	No PIMs Use	p-value
Gender			
Female	73 (57.5)	46 (63)	0.443^a^
Male	54 (42.5)	27 (37)	
**Age,years (median-IQR)**	70 (67–75)	70 (67-74.5)	0.094^b^
Education level*			
< 8 years	77 (60.6)	36 (49.3)	0.12^a^
≥ 8 years	50 (39.4)	37 (50.7)	
**Marital status**			
Single	5 (3.9)	3 (4.1)	0.479^a^
Married	88 (69.3)	56 (76.7)	
Widow	34 (26.8)	14 (19.2)	
**Drug allergy**			
Yes	9 (7.1)	4 (5.5)	0.772^c^
No	118 (92.9)	69 (94.5)	
**History of hospitalization in the last 6 months**			
Yes	28 (22)	20 (27.4)	0.394^d^
No	99 (78)	53 (72.6)	
**Number of comorbidities-ICD 10 (median-IQR)**	2 (1–3)	1 (1–2)	< 0.001^b^
**E00-E89 Endocrine, nutritional and metabolic diseases**	52 (40.9)	24 (32.9)	0.258^a^
E00-E07 Disorders of thyroid gland	9 (7.1)	9 (12.3)	0.322^d^
E08-E13 Diabetes mellitus	48 (37,8)	19 (26)	0.09^a^
**F01-F99 Mental, Behavioral and Neurodevelopmental disorders**	40 (31.5)	7 (9.6)	0.001^d^
**I00–I99 Diseases of the circulatory system**	111 (87.4)	65 (89)	0.906^d^
**J00–J99 Diseases of the respiratory system**	33 (26)	11 (15.1)	0.106^d^
J 44 COPD	12 (9.4)	3 (4.1)	0.264^c^
J 45 Asthma	21 (16.5)	9(12.3)	0.551^d^
**N00–N99 Diseases of the genitourinary system**	18 (14.2)	9 (12.3)	0.879^d^
N18 Chronic Kidney Disease	18 (14.2)	9 (12.3)	0.879^d^
**Number of drugs (mean ± SD)**	4,44 ± 1.82	2.98 ± 1.44	< 0.001^e^
**Polypharmacy (Concurrent use of ≥ 5 drugs)**	58 (45.7)	9 (12.3)	< 0.001^d^
**MRCI score (mean ± SD)**	13.18 ± 5.98	8.68 ± 4.98	< 0.001^e^
MRCI Part A (median-IQR)	5 (3–7)	3 (2–5)	< 0.001^b^
MRCI Part B (mean ± SD)	5.33 ± 2.43	3.79 ± 2.26	< 0.001^e^
MRCI Part C (mean ± SD)	2.53 ± 1.2	1.43 ± 1.1	< 0.001^e^

*The minimum period of compulsory education in Turkey is 8 years. ICD10 International Statistical Classification of Diseases and Related Health Problems 10th Revision, IQR: Interquartile range, COPD: Chronic obstructive pulmonary disease, MRCI: Medication Regimen Complexity Index, PIM: Potentially inappropriate medication, SD: Standard deviation, ^a^ Pearson Chi Square test, ^b^ Mann Whitney U test, ^c^ Fisher’s Exact test, ^d^ Continuity Correction test, ^e^ Student’s T-test

### Medication regimen complexity index (MRCI)

The median of the MRCI score was 11 (IQR, 7–15) (Table 1). Table 4 shows the comparison of the MRCI scores with the socio-demographic and clinical characteristics of the patients. Having less than eight years of education, the presence of polypharmacy and presence of comorbidity (diabetes mellitus, cardiovascular disease, thyroid, chronic obstructive pulmonary disease (COPD), asthma, and mental disorders) were associated with significantly higher MRCI scores (p < 0,05).

**Table 4:** Relationship between MRCI scores and patient characteristics

Characteristics	MRCI scores	p-value
**Overall score (median, IQR)** 11 (7–15)		
**Gender (median, IQR)**		
Female	11 (8–16)	0.209^a^
Male	10 (7–14)	
**Age years (mean ± SD)**		
**< 75**	11.28 ± 6.08	0.236^b^
**≥ 75**	12.54 ± 5.79	
**Education level**		
< 8 years	12.35 ± 6.28	0.031^b^
≥ 8 years	10.49 ± 5.53	
**Marital status (median, IQR)**		
Single	6.5 (5-12.75)	0.125^c^
Married	11 (7–15)	
Widow	12 (8–16)	
**Drug allergy (mean ± SD)**		
No	11.43 ± 6	0.319^b^
Yes	13.15 ± 6.44	
**Polypharmacy (Concurrent use of ≥ 5 drugs) (median, IQR)**	17 (15–21)	< 0.001^a^
**History of hospitalization in the last 6 months (median, IQR)**		
No	11 (7–15)	0.218^a^
Yes	13 (8–16)	
**Comorbidites- ICD 10 (median, IQR)**		
E00-E89 Endocrine, nutritional and metabolic diseases	15 (11–19)	< 0.001^a^
E00-E07 Disorders of thyroid gland	13 (11.5–17.5)	0.018^a^
E08-E13 Diabetes mellitus	15 (11–19)	< 0.001^a^
F01-F99 Mental, Behavioral and Neurodevelopmental disorders	15 (8–19)	0.008^a^
I00–I99 Diseases of the circulatory system	11 (8-15.75)	0.037^a^
J00–J99 Diseases of the respiratory system	16.5 (13–21)	< 0.001^a^
J44 COPD	19 (15–22)	< 0.001^a^
J45 Asthma	15.5 (11.75-19)	< 0.001^a^
N00–N99 Diseases of the genitourinary system	13 (9–15)	0.227^a^
N18 Chronic Kidney Disease	13 (9–15)	0.227^a^

* The minimum period of compulsory education in Turkey is 8 years. MRCI: Medication regimen complexity index, ICD10 International Statistical Classification of Diseases and Related Health Problems 10th Revision, IQR: Interquartile range, SD: Standard deviation, COPD: Chronic obstructive pulmonary disease, ^a^ Mann Whitney U test, ^b^ Student’s T-test ^c^ Kruskal-Wallis test,

## Discussion

In this study, the rate of polypharmacy and MRCI were not found to be high, while the use of PIMs was found to be higher compared to other two scores.

In a study conducted in 10 pharmacies in Argentina, polypharmacy rates were found to be between 20.5% and 47.1% [36]. In a Swiss study of community-dwelling older adults, polypharmacy was 17% [37], and in a study conducted in Singapore, the prevalence of polypharmacy among the elderly living in the community was 14.5% [38]. Although the polypharmacy rate in our study was slightly higher than the polypharmacy rates in the studies [36–38], some studies found polypharmacy much higher than the results of our study [39, 40]. In a study conducted in a pharmacy in Turkey, the rate of polypharmacy was found to be 69% [39]. In the study conducted in the primary health center in Turkey, the rate of polypharmacy was 62.3% [40]. The polypharmacy criterion of the mentioned studies was the use of five or more drugs. Reasons for different polypharmacy rates in studies may include different health care systems, patients’ socioeconomic status, clinical condition, physicians’ prescribing habits, and physician-patient interaction [41]. The high rate of polypharmacy in elderly patients can lead to clinical consequences such as inappropriate drug use, drug-drug interactions, adverse drug reactions, stroke, hospitalization and death [16]. Therefore, patients’ drug use should be monitored, unnecessary drug use should be prevented, and prescriptions should be written by the physician when necessary [42].

A study with Beers Criteria in pharmacies in Lebanon found the use of PIMs at a rate of 45.2%, and benzodiazepines (29%) were the most common examples of PIMs [14]. In a study conducted in geriatric outpatient clinics in India, PIMs were found at a rate of 65%, and the most common PIM as proton pump inhibitors (59.6%) [43]. In a study conducted in China using Beers Criteria, 64.8% of PIMs were used, with proton pump inhibitors (29.15%) and diuretics (16.63%) the most commonly used PIMs. Additionally, the high number of medications was found to be a risk factor associated with PIM [3]. In a study conducted in Saudi Arabia using the Beers Criteria, the use of PIMs was determined at a rate of 57.5%, and the most frequent use of PIMs in gastrointestinal and endocrine group drugs was determined (35.6% and 34.3%, respectively). Polypharmacy and many chronic diseases such as hypertension, diabetes, dyslipidemia, heart failure, ischemic heart failure, chronic renal failure, osteoporosis, and osteoarthritis were found to be PIM-related risk factors. [44]. In a study conducted in a primary care center in Brazil using the Beers Criteria, the prevalence of PIM was found to be 34.5%, and risk factors associated with PIM were illiteracy, black skin color, use of more than four medications per day, and use of medications prescribed by a doctor [45]. In a study conducted in a community health center in Taiwan using the Beers Criteria, the PIM rate was found to be 27.5%. Patients with PIM were significantly older, prescribed more medications, and visited more frequently for acute illnesses than those without PIM [46].The rate of PIMs in our study was found to be higher than in other studies, and the most commonly used PIM groups were diuretics and proton pump inhibitors (21% and 18.5%, respectively). This may be since patients have a low level of knowledge about proton pump inhibitors, so they can easily be bought from pharmacies without a prescription, or it may be due to unnecessary prescriptions by physicians [47]. In our study, similar to other studies, the number of medications used, polypharmacy and comorbidities were significant in the group with PIM compared to those without PIM.

In a study conducted with elderly patients admitted to a pharmacy in Turkey [39], the median MRCI score was 12.5 (7-19.6), and the MRCI A, B, and C scores were 3.0 (1.0–5.0), 8.0 (5.0–13.0), and 1.0 (0.0–3.0), respectively. In addition, in the MRCI validation study conducted in a pharmacy in Turkey [24], the median MRCI value was found to be 12 (7.5–19). The MRCI score was not associated with variables such as age, gender, marital status, education level, as in our study (p > 0,05) [24]. In a study conducted in long-term care facilities in Brazil, the mean MRCI score was calculated as 15.1 ± 9.8, while the mean MRCI A, B, and C scores were 4.6 ± 3, 5.5 (± 3.6), and 4.9 (± 3.7), respectively. Polypharmacy, potential drug-drug interactions, potential inappropriate medication use, and therapeutic duplication were associated with higher MRCI scores [48]. The mean MRCI score of hospitalized elderly patients in Korea was 28.2 ± 14.2, and the MRCI A, B, and C scores were 2.4 ± 1.7, 11.8 ± 6.0, and 14.0 ± 8.1, respectively [49]. The mean MRCI score for both adults and the elderly in primary care in Brazil was 8.5. The number of drugs, polypharmacy, potential drug-related problems, and clinical conditions (cardiovascular and endocrine diseases) were significantly associated with higher MRCI [50]. In a study conducted in a primary care center in Brazil, the median MRCI score was found to be 12, and no correlation was found between MRCI scores and age, gender, cognition, basic and instrumental activities of daily living [51]. In a study in a long-term care facility in South Australia, the MRCI median was found to be 43.5, and chronic pulmonary disease, diabetes, and congestive heart failure were associated with higher regimen complexity [30]. Similarly, in our study, MRCI scores were found to be significantly higher with polypharmacy and clinical conditions (diabetes, thyroid, cardiovascular disease, COPD, asthma, mental disorders). Additionally, unlike other studies, in our study, low education level was found to be associated with high MRCI scores.

The threshold of the MRCI score is not clear. In one study, a median MRCI score of less than 16.5 was associated with lower medication complexity in primary care [51]. The MRCI score of our study was parallel to studies conducted in pharmacies. However, our MRCI score was low compared to studies conducted in hospitals and long-term care facilities. This may be since we include elderly patients who come to the pharmacy for any reason, and that it includes less vulnerable elderly groups such as hospitals and long-term care facilities. In addition, it may be caused by missing medication instructions during patient interviews and medications that patients did not specify. In our study, there is a statistical relationship between the presence of PIMs and a high MRCI score, similar to the previous study [39].

In our study, PIM use was found to be high. Community pharmacists play an important and growing role in providing pharmaceutical care to patients [52]. Pharmacist review has the potential to simplify patients’ drug regimens and reduce inappropriate drug use [19]. Although only physicians have the authority to write prescriptions in Turkey [53], pharmacists can also contribute to the prevention of drug-related problems by communicating with the physician and informing the patient about inappropriate drug use. However, we do not know what level of knowledge and attitude of community pharmacists in Turkey about these issues. Knowledge, attitudes and practices of pharmacists, both in Turkey and around the world, about the criteria for PIM use in the elderly, such as Beers, should be investigated. For this to be routinely implemented in community pharmacies, pharmacists’ awareness of polypharmacy, simplification of complex regimens and inappropriate drug use needs to be increased. Also, continuous training of pharmacists on these issues is required. Future studies need examine the impact of community pharmacists’ simplification of complex medication regimens and reduction of inappropriate drug use on clinical outcomes such as fall risk and hospitalization.

This study had some limitations. Data were collected with only one senior student over a period of time, and only patients who agreed to participate in the study were included. Therefore, the number of elderly patients included in the study was limited and sufficient sample size may not have been achieved. Additionally, the study had limitations such as random selection of the study area, use of the interview method, and selection of participants through convenience sampling. Missing medication instructions may have been recorded during the patient interview. In addition, patients were not assessed for adherence and adverse drug reactions.

## Conclusions

According to the results of our study, it was found that the elderly patients who came to the pharmacy had low MRCI scores, but had high PIMs use. Community pharmacists have an important role in identifying inappropriate drug use, so they should be trained to develop skills in identifying and reducing PIMs in older patients.

## Data Availability

All data generated or analyzed during this study are included in this published article.

## References

[1] Jirón M, Pate V, Hanson LC, Lund JL, Jonsson Funk M, Stürmer T (2016). Trends in prevalence and determinants of potentially inappropriate prescribing in the United States: 2007 to 2012. J Am Geriatr.

[2] Masnoon N, Shakib S, Kalisch-Ellett L, Caughey GE (2017). What is polypharmacy? A systematic review of definitions. BMC Geriatr.

[3] He D, Zhu H, Zhou H, Dong N, Zhang H (2021). Potentially inappropriate medications in Chinese older adults: a comparison of two updated Beers criteria. Int J Clin Pharm.

[4] Curtin D, Gallagher PF, O’Mahony D (2019). Explicit criteria as clinical tools to minimize inappropriate medication use and its consequences. Ther Adv Drug Saf.

[5] American Geriatrics Society (2019). 2019 updated AGS Beers Criteria® for potentially inappropriate medication use in older adults. J Am Geriatr Soc.

[6] Beers MH, Ouslander JG, Rollingher I, Reuben DB, Brooks J, Beck JC (1991). Explicit criteria for determining inappropriate medication use in nursing home residents. Arch Intern Med.

[7] American Geriatrics Society. 2023 updated AGS Beers Criteria® for potentially inappropriate medication use in older adults. J Am Geriatr Soc. 2023. 10.1111/jgs.18372 PMC12478568 37139824

[8] Hagstrom K, Nailor M, Lindberg M, Hobbs L, Sobieraj DM (2015). Association between potentially inappropriate medication use in elderly adults and hospital-related outcomes. J Am Geriatr Soc.

[9] Praxedes MFS, Pereira GCS, Lima CFM, Santos DBd, Berhends JS (2021). Prescribing potentially inappropriate medications for the elderly according to Beers Criteria: systematic review. Cien Saude Colet.

[10] Wallace E, McDowell R, Bennett K, Fahey T, Smith SM (2017). Impact of potentially inappropriate prescribing on adverse drug events, health related quality of life and emergency hospital attendance in older people attending general practice: a prospective cohort study. J Gerontol A Biol Sci Med Sci.

[11] Chahine B (2020). Potentially inappropriate medications prescribing to elderly patients with advanced chronic kidney by using 2019 American Geriatrics Society Beers Criteria. Health Sci Rep.

[12] Xu Z, Liang X, Zhu Y, Lu Y, Ye Y, Fang L et al. Factors associated with potentially inappropriate prescriptions and barriers to medicines optimisation among older adults in primary care settings: a systematic review. Fam Med Community Health. 2021;9(4). 10.1136/fmch-2021-001325 PMC8603289 34794961

[13] Alhmoud E, Khalifa S, Bahi AA (2015). Prevalence and predictors of potentially inappropriate medications among home care elderly patients in Qatar. Int J Clin Pharm.

[14] Zeenny R, Wakim S, Kuyumjian Y-M. Potentially inappropriate medications use in community-based aged patients: a cross-sectional study using 2012 Beers criteria. Clin Interv Aging. 2017:65–73. 10.2147/CIA.S87564 PMC5221543 28115835

[15] Advinha AM, de Oliveira-Martins S, Mateus V, Pajote SG, Lopes MJ (2014). Medication regimen complexity in institutionalized elderly people in an aging society. Int J Clin Pharm.

[16] Wastesson JW, Morin L, Tan EC, Johnell K (2018). An update on the clinical consequences of polypharmacy in older adults: a narrative review. Expert Opin Drug Saf.

[17] Advinha AM, Lopes MJ, de Oliveira-Martins S (2017). Assessment of the elderly’s functional ability to manage their medication: a systematic literature review. Int J Clin Pharm.

[18] Silva C, Ramalho C, Luz I, Monteiro J, Fresco P (2015). Drug-related problems in institutionalized, polymedicated elderly patients: opportunities for pharmacist intervention. Int J Clin Pharm.

[19] Falch C, Alves G (2021). Pharmacists’ role in older adults’ medication regimen complexity: a systematic review. Int J Environ Res Public Health.

[20] Kelley SO. Measurement of the complexity of medication regimens of the elderly. University of Missouri-Columbia; 1988.

[21] DiIorio C, Yeager K, Shafer PO, Letz R (2003). The Epilepsy medication and treatment complexity index: reliability and validity testing. J Neurosci Nurs.

[22] DiIorio C, McDonnell M, McCarty F, Yeager K (2006). Initial testing of the antiretroviral medication complexity index. J Assoc Nurses AIDS Care.

[23] George J, Phun Y-T, Bailey MJ, Kong DC, Stewart K (2004). Development and validation of the medication regimen complexity index. Ann Pharmacother.

[24] Okuyan B, Babi B, Sancar M, Ay P, Yücel E, Yücel A (2016). Validation of the Turkish version of medication regimen complexity index among elderly patients. J Eval Clin Pract.

[25] Alves-Conceição V, Rocha KSS, Silva FVN, Silva RdOS, Cerqueira-Santos S, Nunes MAP (2020). Are clinical outcomes associated with medication regimen complexity? A systematic review and meta-analysis. Ann Pharmacother.

[26] Abou-Karam N, Bradford C, Lor KB, Barnett M, Ha M, Rizos A (2016). Medication regimen complexity and readmissions after hospitalization for Heart Failure, acute Myocardial Infarction, Pneumonia, and Chronic Obstructive Pulmonary Disease. SAGE Open Med.

[27] Willson MN, Greer CL, Weeks DL (2014). Medication regimen complexity and hospital readmission for an adverse drug event. Ann Pharmacother.

[28] Kuo SZ, Haftek M, Lai JC (2017). Factors associated with medication non-adherence in patients with end-stage Liver Disease. Dig Dis Sci.

[29] Wimmer BC, Johnell K, Fastbom J, Wiese MD, Bell JS (2015). Factors associated with medication regimen complexity in older people: a cross-sectional population-based study. Eur J Clin Pharmacol.

[30] Herson M, Bell J, Tan E, Emery T, Robson L, Wimmer B (2015). Factors associated with medication regimen complexity in residents of long-term care facilities. Eur Geriatr Med.

[31] Bužančić I, Kummer I, Držaić M, Ortner Hadžiabdić M (2022). Community-based pharmacists’ role in deprescribing: a systematic review. Br J Clin Pharmacol.

[32] Yakar M, Özgür EM. Türkiye’de Nüfus Yaşlanması, Yerel Düzeyde Tehlike Çanları Çalıyor. Coğrafya Derg. 2022(44):231–50.

[33] TÜİK. (2022). İstatistiklerle Yaşlılar, 2022. Available from: https://data.tuik.gov.tr/Bulten/Index?p=%C4%B0statistiklerle-Ya%C5%9Fl%C4%B1lar-2022-49667&dil=1. Accessed 19 Aug 2023.

[34] Raosoft Inc. (2004) RaoSoft® sample size calculator. Available from: http://www.raosoft.com/samplesize.html. Accessed 01 Jun 2022.

[35] Pazan F, Wehling M (2021). Polypharmacy in older adults: a narrative review of definitions, epidemiology and consequences. Eur Geriatr Med.

[36] Chiapella LC, Montemarani Menna J, Mamprin ME (2018). Assessment of polypharmacy in elderly patients by using data from dispensed medications in community pharmacies: analysis of results by using different methods of estimation. Int J Clin Pharm.

[37] Blozik E, Rapold R, von Overbeck J, Reich O (2013). Polypharmacy and potentially inappropriate medication in the adult, community-dwelling population in Switzerland. Drugs Aging.

[38] Tan YW, Suppiah S, Bautista MAC, Malhotra R (2019). Polypharmacy among community-dwelling elderly in Singapore: prevalence, risk factors and association with medication non-adherence. Proc Singap Healthc.

[39] Sayın Z, Sancar M, Özen Y, Okuyan B (2022). Polypharmacy, potentially inappropriate prescribing and medication complexity in Turkish older patients in the community pharmacy setting. Acta Clin Belg.

[40] Öztürk GZ, Ardic C, Toprak D. Frequency of polypharmacy and use of potentially inappropriate medications in the elderly. Turk J Geriatr. 2017;20(4).

[41] Hovstadius B, Petersson G (2012). Factors leading to excessive polypharmacy. Clin Geriatr Med.

[42] Halli-Tierney AD, Scarbrough C, Carroll D (2019). Polypharmacy: evaluating risks and deprescribing. Am Fam Physician.

[43] Anand P, Katyal J, Dey AB, Gupta YK (2022). Characterization of potentially inappropriate medications use in Indian elderly population and their impact on quality of life using Beers criteria. Aging Med.

[44] Alhawassi TM, Alatawi W, Alwhaibi M (2019). Prevalence of potentially inappropriate medications use among older adults and risk factors using the 2015 American Geriatrics Society Beers criteria. BMC Geriatr.

[45] Oliveira MG, Amorim WW, de Jesus SR, Rodrigues VA, Passos LC (2012). Factors associated with potentially inappropriate medication use by the elderly in the Brazilian primary care setting. Int J Clin Pharm.

[46] Lin Y-J, Peng L-N, Chen L-K, Lin M-H, Hwang S-J (2011). Risk factors of potentially inappropriate medications among older patients visiting the community health center in rural Taiwan. Arch Gerontol Geriatr.

[47] Luo H, Fan Q, Bian T, Li X, Chen K, Zhang Q (2019). Awareness, attitude and behavior regarding Proton pump inhibitor among medical staff in the Southwest of China. BMC Health Serv Res.

[48] Alves-Conceição V, Silva DTd S, VLd S, EGd, Santos LMC, Lyra DP (2017). Evaluation of pharmacotherapy complexity in residents of long-term care facilities: a cross-sectional descriptive study. BMC Pharmacol Toxicol.

[49] Lee S, Jang J, Yang S, Hahn J, Min KL, Jung E (2019). Development and validation of the Korean version of the medication regimen complexity index. PLoS One.

[50] Ferreira JM, Galato D, Melo AC. Medication regimen complexity in adults and the elderly in a primary healthcare setting: determination of high and low complexities. Pharm Pract. 2015;13(4). 10.18549/PharmPract.2015.04.659 PMC4696124 26759621

[51] Pantuzza LL, Ceccato MGB, Silveira MR, Pinto IV, Reis AMM (2018). Validation and standardization of the Brazilian version of the Medication Regimen Complexity Index for older adults in primary care. Geriatr Gerontol Int.

[52] Van Mil JF, Schulz M (2006). A review of pharmaceutical care in community pharmacy in Europe. Harv Health Policy Rev.

[53] Ergün Y, Aykan DA (2019). Akılcı İlaç Kullanımında Genel İlkeler. Arşiv Kaynak Tarama Derg.

